# LIFESTYLE AND ANTHROPOMETRIC INDICATORS HAVE GREATER ASSOCIATIONS WITH STEPS/DAY IN BOYS THAN IN GIRLS

**DOI:** 10.1590/1984-0462/2021/39/2019413

**Published:** 2020-12-14

**Authors:** Eduardo Rossato de Victo, Gerson Ferrari, Carlos André Miranda Pires, Dirceu Solé, Timóteo Leandro Araújo, Peter Todd Katzmarzyk, Victor Keihan Rodrigues Matsudo

**Affiliations:** a Allergy, Clinical Immunology and Rheumatology Discipline, Pediatrics Departament, Universidade Federal de São Paulo, São Paulo, SP, Brazil.; b Laboratory of Sciences of Physical Activity, Sports and Health, Faculty of Medical Sciences, Universidad de Santiago de Chile, Santiago, Chile.; c Center for research in Neuropsychology and Cognitive and Behavioral Intervention, Faculty of Psychology and Educational Sciences, Universidade de Coimbra, Portugal.; d Study Center of the Physical Fitness Laboratory of São Caetano do Sul, São Caetano do Sul, SP, Brazil.; e Pennington Biomedical Research Center, Baton Rouge, LA, United States.

**Keywords:** Motor activity, Lifestyle, Body composition, Public health, Pediatrics, Students, Atividade motora, Estilo de vida, Composição corporal, Saúde pública, Pediatria, Estudantes

## Abstract

**Objective:**

To verify the association of lifestyle, anthropometric, sociodemographic, family and school environment indicators with the number of steps/day in children.

**Methods:**

The sample consisted of 334 children (171 boys) from nine to 11 years old. Participants used the Actigraph GT3X accelerometer to monitor the number of steps/day, moderate to vigorous physical activity (MVPA) and sedentary time (ST) for seven consecutive days. Height, body weight, body mass index (BMI), waist circumference (WC), and body fat were also measured. Lifestyle indicators such as diet, environment, neighborhood, and parental schooling level were obtained with questionnaires. For the identification of variables associated to the number of steps/day, multiple linear regression models were used.

**Results:**

The mean steps/day of boys and girls were statistically different (10,471 versus 8,573; p<001). Among boys, the variables associated to the number of steps/day were: MVPA (β=0.777), ST (β=-0.131), BMI (β=-0.135), WC (β=-0.117), and BF (β=-0.127). Among girls, the variables associated to the number of steps/day were: MVPA (β=0.837), ST (β=-0.112), and parents’ educational level (β=0.129).

**Conclusions:**

Lifestyle indicators, body composition variables and parental educational level influence the number of steps/day of children, and MVPA and ST are common for both sexes.

## RESUMO

**Objetivo:**

Verificar a associação dos indicadores de estilo de vida, antropométricos, sociodemográficos, ambiente familiar e escolar com a quantidade de passos/dia em crianças.

**Métodos:**

A amostra constituiu-se de 334 crianças (171 meninos) de 9 a 11 anos. Os participantes utilizaram o acelerômetro Actigraph GT3X para monitorar a quantidade de passos/dia, a atividade física moderada a vigorosa (AFMV) e o tempo sedentário (TS) durante sete dias consecutivos. Estatura, massa corporal, índice de massa corpórea (IMC), circunferência de cintura (CC) e gordura corporal também foram mensurados. Indicadores de estilo de vida, como dieta, ambiente, vizinhança e nível de escolaridade dos pais, foram obtidos por questionários. Para identificar as variáveis associadas à quantidade de passos/dia, utilizaram-se modelos de regressão linear múltipla.

**Resultados:**

As médias de passos/dia dos meninos e das meninas foram estatisticamente diferentes (10.471 versus 8.573; p<0,001). Nos meninos, as variáveis associadas à quantidade de passos/dia foram: AFMV (β=0,777), TS (β=-0,131), IMC (β=-0,135), CC (β=-0,117) e gordura corporal (β=-0,127). Já entre as meninas, as variáveis associadas à quantidade de passos/dia foram: AFMV (β=0,837), TS (β=-0,112) e nível educacional dos pais (β=0,129).

**Conclusões:**

Indicadores de estilo de vida, variáveis de composição corporal e nível educacional dos pais influenciaram a quantidade de passos/dia das crianças. A AFMV e o TS foram comuns para ambos os sexos.

## INTRODUCTION

The number of steps/day is a simple measure that quantifies the total daily volume of physical activity (PA).^1^ As it is a basic and fundamental measure of human locomotion, its use is easy to measure and translate scientific results for public health messages. Besides that, its motivational power facilitates behavioral changes.^2^

There is growing interest in using PA recommendations based on steps/day, mainly because they have associations with physical, cardiac and metabolic health, in addition to obesity.^1^ ^,^ ^2^ ^,^ ^3^ ^,^ ^4^ The World Health Organization (WHO) recommends that children and adolescents should reach 12 thousand steps/day or accumulate 60 min/day of moderate to vigorous physical activity (MVPA).^5^ For preventing overweight and obesity, a multicenter study carried out in the United States, Sweden, and Australia proposed different values for boys and girls (15 thousand and 12 thousand steps/day).^6^ In Brazil, the proposed values are lower: 10,500 (boys) and 8,500 (girls) steps/day.^7^

Accumulating steps/day or PA, from childhood to adulthood, has short- and long-term health benefits.^8^ However, the associated factors of PA during childhood need to be well understood to design effective intervention strategies. PA is influenced by complex and diverse factors, and theories of behavioral models are used to guide the selection of variables.^9^ Integrating theories in an ecological model - including anthropometry (i.e., body weight and waist circumference), individual behavior (i.e., sedentary behaviors, screen time, transportation to school, and sleep), and family environment (for example, family income and parental education level) - is common.^10^ ^,^ ^11^ This approach uses a comprehensive framework to explain PA, proposing that the associated factors at all levels are contributing factors.

Despite investigating possible factors related to PA, there are still gaps in relation to steps/day, mainly due to the lack of research with objective instruments, such as accelerometry, which requires a combination of financial resources and technological knowledge, challenging researchers from low and middle income countries.^12^ The use of accelerometers is a good strategy to measure the number of steps/day, as it produces objective information with high agreement and validation values.^13^ ^,^ ^14^ Given this, the objective of the present study was to verify the lifestyle, anthropometric, sociodemographic, family, and school environment indicators associated to the number of steps/day of children participating in the International Study of Childhood Obesity, Lifestyle and Environment (ISCOLE), Brazil. We sought to verify the possibility of a significant association between lifestyle, anthropometric, sociodemographic and environmental indicators, and children’s number of steps/day.

## METHOD

The present study is cross-sectional, with analysis of the Brazilian data from ISCOLE, which is a multicenter study developed in 12 countries. Details about ISCOLE were described by Katzmarzyk et al.^15^ In Brazil, data were obtained in São Caetano do Sul City, between 2012 and 2013. In 2013, it had 149,263 inhabitants, of which 1,557 were children aged 10 years old.^16^ The city stands out for having the highest human development index (HDI) in Brazil.^17^ Details on school selection and sample calculation were shown by Ferrari et al.^11^ Children and at least one parent or legal guardian signed the Free and Informed Consent Form. The project was approved by the Research Ethics Committee of Universidade Federal de São Paulo.^11^

A total of 564 children who met the inclusion criteria participated in the study (between nine and 11 years old, regularly enrolled in a school in the city, and no clinical or functional conditions that limited the practice of PA). Invalid accelerometry data or incomplete information was excluded from the study. Thus, the total sample was composed of 334 children.

In order to monitor the number of steps/day, MVPA, and sedentary time (TS), Actigraph GT3X accelerometer (ActiGraph, Ft. Walton Beach, USA) was used. The device was placed on the waist with an elastic belt, on the right axillary midline. Children were encouraged to use the accelerometer 24 hours a day for at least seven days (plus one day of initial familiarization and the morning of the last day), including two weekend days. Children should remove the accelerometer only for water activities and bathing. The minimum amount of accelerometer data considered acceptable for the analysis was four days (including at least one weekend day), with at least 10 hours/day of usage time, after removal at sleep time.^18^ Blocks of 20 consecutive minutes with zero *count* were considered as not using the device and were eliminated from analysis. Version 5.6 of ActiLife *software* was used to verify data.

Information was collected at a sampling rate of 80 Hz, in one-second periods, later incorporated into 15-second cycles.^19^ Cut points were classified as follows: ST (≤25 counts/15 seconds), moderate PA (≥574 to 1,002 counts/15 seconds), vigorous PA (≥1,003 counts/15 seconds). The total MVPA was considered ≥574 counts/15 seconds.^19^

Height was measured with a portable Seca 213 stadiometer (Seca^®^, Hamburg, Germany), with the child’s head on Frankfurt plane and no shoes.^15^ Body mass and body fat (BF) percentage were measured with a Tanita SC-240 scale (Arlington Heights, IL, USA), portable body composition analyzer, after removing heavy items from the pocket, shoes and socks.^20^ Two measurements were obtained, and the mean was used (a third measurement was obtained when the first two gave a difference greater than 0.5 kg or 2% for body mass and fat percentage, respectively). Body mass index (BMI; kg/m^2^) was calculated based on the WHO growth curve references.^21^ Children were classified as: underweight (<−2 standard deviation - SD), eutrophic (−2 SD to 1 SD), overweight (> 1 SD to 2 SD), and obesity (>2 SD).21 Waist circumference (WC) was measured with an inelastic anthropometric tape between the rib and the iliac crest.^15^

For obtaining data on food consumption, sedentary behavior, and screen time, the Diet and Lifestyle Questionnaire was applied.^15^ Food consumption was analyzed by evaluating 23 items during a usual week. To identify existing dietary patterns, principal component analysis (PCA) was used. PCA was performed with orthogonal varimax transformation to force non-correlation and improve interpretation. Two factors were identified: unhealthy diet pattern (sweets, fast food, soft drinks, etc.) and healthy diet pattern (fruits, vegetables, greens, among others).^15^ Both scores were analyzed separately and treated as continuous variables. The highest values for each score represent an unhealthy or healthy diet pattern, respectively. Children also reported the frequency of eating breakfast.

Children were asked about the number of hours they watched television, played video games, or used the computer on weekdays and weekends. Total screen time was calculated by the sum of individual activities.^11^ Children classified the quantity and quality of sleep as: very bad, bad, good, or very good.^15^

For the type of transport to school, the answers were: by walking; bicycle, skates, skateboard or scooter; bus, train, subway, boat; car/motorcycle; other. Responses were categorized into active or passive transport. Adolescents also answered the time spent during the journey to school: <5; 5-15; 16-30; 31-60; >60 minutes.

The Neighborhood and Home Environment Questionnaire was completed by the children’s parents or legal guardians. The questionnaire included questions related to the child’s health history, the environment they lived in, their parents’ professional situation, their annual family income, and their parents’ educational level.^15^ The annual family income (R$) was classified into four categories: <R$ 19,620; R$ 19,620 to 32,700; R$ 32,701 to 58,860; >R$ 58,860. The combined educational level of parents (highest level of any parent) was classified as: incomplete high school, complete high school or undergraduate/graduate education.^15^

The School Environment Questionnaire measured information related to the child’s school, providing information on the type of administration (public or private), PA policies, healthy eating, and the number of physical education classes in their curriculum.^15^ For adapting questionnaires, three health professionals were invited to participate in the stage. Detailed information about the questionnaires was sent, with meetings held separately with each professional, until a consensus was reached regarding the composition of questionnaires, their questions, the options for answers, as well as the ways for analyzing results.

Analyzes were stratified by sex by the difference in the number of steps/day between boys and girls. Variables were categorized by means and standard deviation, or absolute and relative frequencies. Differences between groups (p<0.05) were assessed with Student’s t-test, for independent samples, and the chi-square test.

In order to identify the variables associated to the number of steps, linear regression models were used. In a first phase, simple models were performed, adjusted for sex and skin color. The variables that showed significant values (p<0.10) were later included in multiple models, also adjusted for sex and skin color. In these models, the stepwise method was used to exclude non-significant variables. Thus, only variables significantly associated to the number of steps remained in the final models, considering the descriptive level of the test p<0.05. As for the assumptions of regression models, the normality of the dependent variable was validated with the Kolmogorov-Smirnov test (p>0.20). The normality and homoscedasticity of the model residues were also tested and validated. For assessing possible effects of multicollinearity between independent variables, correlations and the variance inflation factor (VIF) were analyzed. VIF values>5 were considered indicators of problems in estimating coefficients by multicollinearity.

As to the diet and lifestyle questionnaire, the reliability of healthy and unhealthy eating scales was assessed with Cronbach’s *alpha*. The values obtained, 0.760 and 0.741, respectively, are indicators of good reliability for both scales. The scales’ scores vary from 1-7 - the higher the score, the greater the frequency of consumption of healthy and unhealthy foods. Analyzes were performed using the Statistical Package for the Social Sciences (SPSS) software, version 22. 0.

## RESULTS

The sample included 334 students (171 boys) with an average of 10.4 years old. More than half were from families with an annual income of up to R$ 32,700, of which 65.5% of mothers worked full time, and more than half of parents completed high school. The percentage of students who attended schools with PA policies was higher in girls than in boys (Table 1).

**Table 1:** Sociodemographic and environmental characteristics (mean [standard deviation] or n [%]) according to sex.

	Male (n=171)	Female (n=163)	p-value
Sociodemographic			
Age (years old)	10.4 (0.5)	10.4 (0.5)	0.822*
Skin color			
White/Caucasian	125 (73.1)	128 (78.5)	0.096**
Black	13 (7.6)	11 (6.7)	
Mixed	28 (16.4)	14 (8.6)	
Other	5 (2.9)	10 (6.1)	
Annual family income (R$)			
Up to 19,620	59 (34.5)	58 (35.6)	0.155**
From 19,621 to 32,700	53 (31.0)	34 (20.9)	
From 32,701 to 58,860	37 (21.6)	42 (25.8)	
More than 58,860	22 (12.9)	29 (17.8)	
Mother’s professional situation			
Part-time employed or less	95 (55.6)	78 (47.9)	0.159**
Full-time employed	76 (44.4)	85 (52.1)	
Father’s professional situation			
Part-time employed or less	59 (34.5)	56 (34.4)	0.977**
Full-time employed	112 (65.5)	107 (65.6)	
Paents’ combined educational level			
Incomplete high school	37 (21.6)	34 (20.9)	0.982**
Complete high school	96 (56.1)	93 (57.1)	
Undergraduation or graduation	38 (22.2)	36 (22.1)	
Family environment			
Number of siblings	1.3 (1.1)	1.3 (1.1)	0.922*
Number of TVs at home	2.3 (1.0)	2.3 (0.9)	0.471*
TV in the bedroom			
No	45 (26.3)	40 (24.5)	0.710**
Yes	126 (73.7)	123 (75.5)	
Number of cars at home	1.0 (0.8)	1.0 (0.8)	0.781*
School environment			
Type of school			
Public	167 (97.7)	158 (96.9)	0.681**
Private	4 (2.3)	5 (3.1)	
School with physical activity policies			
No	88 (51.5)	65 (39.9)	0.034**
Yes	83 (48.5)	98 (60.1)	
School with healthy eating policies			
No	99 (57.9)	83 (50.9)	0.201**
Yes	72 (42.1)	80 (49.1)	

Results presented as mean (standard deviation) or n (%); *Student’s t-test for independent samples for comparisons of means and standard deviation; **chi-square test for comparisons of frequency and percentage; SD: standard deviation; TV: television.

There were no significant differences in the unhealthy eating scale score, but boys have a higher average than girls for healthy eating. About 40% of students actively went to school. On average, boys spent 4.1 hours/day watching television, playing video games, or using computers, higher than that of girls (3.6 hours/day). The number of minutes of PA (moderate, vigorous, and MVPA) was significantly higher in boys than in girls. On average, boys perform more steps/day (p<0.001) than girls. ST was higher in girls (p=0.011) when compared to boys. The percentage of BF was higher in girls than in boys (p<0.001). No differences were found between them regarding WC, height, body mass, and BMI. In the categorized BMI, there was a significant difference (p=0.016), and more than half of students were overweight or obese (Table 2).

**Table 2:** Behavioral characteristics (mean [standard deviation] or n [%]), lifestyle and anthropometry according to sex.

	Male (n=171)	Female (n=163)	p-value
Eating			
Healthy eating score (scale from 1 to 7)	3.1 (0.9)	2.8 (0.8)	0.033*
Unhealthy eating score (scale from 1 to 7)	3.8 (1.2)	3.8 (1.1)	0.650*
Breakfast (days/week)	5.5 (2.1)	5.00 (2.2)	0.052*
Commuting to school			
Type of transport			
Passive	105 (61.4)	94 (57.7)	0.487**
Active	66 (38.6)	69 (42.3)	
Time of transport			
≤15 minutes	102 (59.6)	119 (73.0)	0.036**
>15 and ≤30 minutes	40 (23.4)	25 (15.3)	
>30 minutes	29 (17.0)	19 (11.7)	
Screen time (hours/day)			
Total time	4.1 (2.2)	3.6 (2.0)	0.068*
TV time	2.3 (1.4)	2.3 (1.3)	0.667*
Video game or computer time	1.7 (1.3)	1.4 (1.2)	0.009*
Sleep			
Quality			
Bad/very bad	7 (4.1)	9 (5.5)	0.541**
Good/very good	164 (95.9)	154 (94.5)	
Quantity			
Bad/very bad	10 (5.8)	7 (4.3)	0.518**
Good/very good	161 (94.2)	156 (95.7)	
Physical activity			
Physical education classes (days/week)	2.1 (1.0)	2.1 (0.8)	0.449*
MPA (min/day)	47.8 (15.0)	34.3 (12.9)	<0.001*
VPA (min/day)	22.7 (12.6)	12.7 (6.7)	<0.001*
MVPA (min/day)	70.5 (25.8)	46.9 (18.6)	<0.001*
Sedentary time (min/day)	491.1 (68.7)	510.2 (67.2)	0.011 *
Number of steps/day	10,470.6 (2,666.6)	8,573.2 (2,266.9)	<0.001*
Body fat (%)	21.3 (9.6)	25.8 (9.0)	<0.001*
Waist circumference (cm)	67.9 (11.7)	67.0 (9.8)	0.483*
Height (cm)	143.2 (7.1)	144.2 (8.2)	0.236*
Body mass (kg)	41.7 (12.9)	42.3 (12.2)	0.617*
Body mass index (kg/m^2^)	20.0 (4.7)	20.1 (4.5)	0.800*
Body mass index - categorical			
Underweight	3 (1.8)	1 (0.6)	0.016**
Normal weight	82 (48.0)	76 (46.6)	
Overweight	30 (17.5)	50 (30.7)	
Obesity	56 (32.7)	36 (22.1)	

Results presented as mean (standard deviation) or n (%); *Student’s t-test for independent samples for comparisons of means and standard deviation; **chi-square test for comparisons of frequency and percentage; TV: television; MPA: moderate physical activity; VPA: vigorous physical activity; MVPA: moderate to vigorous physical activity; min: minutes; SD: standard deviation.

Tables 3 and 4 show the results of simple regression models, adjusted for age and skin color for each sex. In this model, MVPA and sedentary time achieved significant results for both sexes. Significant variables (p<0.10) were included in multiple regression models (Table 5).

**Table 3:** Simple linear regression models - boys (n=171).

	Non-standardized coefficient B	Standardized coefficient β	p-value
Sociodemographic, family and school characteristics			
Family income (reference: up to R$ 19,620)			
From R$ 19,621 to R$ 32,700	-294.2	-0.051	0.569
From R$ 32,701 to R$ 58,860	-604.8	-0.094	0.290
More than R$ 58,860	-967.1	-0.122	0.154
Mother’s professional situation (reference: part-time or less)			
Full-time employed	553.7	0.103	0.176
Father’s professional situation (reference: part-time or less)			
Full-time employed	576.0	0.103	0.194
Parents’ educational level (reference: incomplete high school)			
Complete high school	276.3	0.052	0.595
Undergraduation or graduation	65.9	0.010	0.916
Number of siblings	-34.0	-0.014	0.855
Number of TVs at home	222.9	0.081	0.301
TV in the bedroom - yes (reference: no)	-535.9	-0.089	0.250
Type of school (reference: public)			
Private school	-147.2	-0.008	0.913
School with physical activity policies - yes (reference: no)	-290.7	-0.055	0.491
School with healthy eating policies - yes (reference: no)	237.9	0.044	0.590
Behavioral characteristics and physical activity			
Healthy eating score (scale from 1 to 7)	-9.5	-0.003	0.965
Unhealthy eating score (scale from 1 to 7)	-131.6	-0.060	0.441
Breakfast (days/week)	-40.0	-0.031	0.694
Type of transport to school (reference: passive)			
Active	153.2	0.028	0.717
Time of transport to school (reference: ≤15 minutes)			
>15 and ≤30 minutes	-151.8	-0.024	0.763
>30 minutes	-730.9	-0.103	0.203
Screen time (hours/day)	-124.8	-0.101	0.187
Quality of sleep (reference: bad/very bad)			
Good/very good	2016.2	0.150	0.051
Quantity of sleep (reference: bad/very bad)			
Good/very good	578.1	0.051	0.508
Physical education classes (days/week)	329.3	0.131	0.096
MVPA (min/day)	93.1	0.900	<0.001
Sedentary time (min/day)	-22.0	-0.566	<0.001
Anthropometric characteristics			
Body fat (%)	-98.4	-0.352	<0.001
Waist circumference (cm)	-84.9	-0.372	<0.001
Body mass index (kg/m^2^)	-192.0	-0.339	<0.001

Simple regression models adjusted for age and skin color - dependent variable: number of steps/day; MVPA: moderate to vigorous physical activity; min: minutes; TV: television.

**Table 4:** Simple linear regression models - girls (n=163).

	Non-standardized coefficient B	Standardized coefficient β	p-value
Sociodemographic, family and school characteristics			
Family income (reference: up to R$ 19,620)			
From R$ 19,621 to R$ 32,700	-369.0	-0.066	0.429
From R$ 32,701 to R$ 58,860	-963.9	-0.187	0.032
More than R$ 58,860	-1,505.0	-0.255	0.003
Mother’s professional situation (reference:part-time or less)			
Full-time employed	354.8	0.078	0.313
Father’s professional situation (reference: part-time or less)			
Full-time employed	-685.3	-0.144	0.067
Parents’ educational level (reference: incomplete high school)			
Complete high school	-149.4	-0.033	0.738
Undergraduation or graduation	-1,371.1	-0.252	0.012
Number of siblings	264.8	0.128	0.097
Number of TVs at home	-539.3	-0.227	0.004
TV in the bedroom - yes (reference: no)	-756.5	-0.144	0.060
Type of school (reference: public)			
Private school	-1,352.9	-0.103	0.180
School with physical activity policies - yes (reference: no)	-684.3	-0.148	0.061
School with healthy eating policies - yes (reference: no)	-508.0	-0.112	0.180
Behavioral characteristics and physical activity			
Healthy eating score (scale from 1 to 7)	66.0	0.023	0.769
Unhealthy eating score (scale from 1 to 7)	141.2	0.071	0.361
Breakfast (days/week)	-62.2	-0.061	0.438
Type of transport to school (reference: passive)			
Active	1,008.4	0.220	0.004
Time of transport to school (referebce: ≤15 minutes)			
>15 and ≤30 minutes	-189.2	-0.030	0.704
>30 minutes	57.1	0.008	0.917
Screen time (hours/day)	-125.7	-0.114	0.148
Quality of sleep (reference: bad/very bad)			
Good/very good	-900.8	-0.091	0.239
Quantity of sleep (reference: bad/very bad)			
Good/very good	-1,102.8	-0.099	0.198
Physical education classess (days/week)	-53.3	-0.020	0.797
MVPA (min/day)	107.7	0.886	<0.001
Sedentary time (min/day)	-17.8	-0.528	<0.001
Anthropometric characteristics			
Body fat (%)	-11.4	-0.045	0.564
Waist circumference (cm)	-10.7	-0.047	0.552
Body mass index (kg/m^2^)	-20.2	-0.040	0.603

Simple regression models adjusted for age and skin color - dependent variable: number of steps/day; MVPA: moderate to vigorous physical activity; min: minutes; TV: television.

**Table 5:** Multiple linear regression models for each sex.

Boys	Non-standardized coefficient B	Standardized coefficient β	p-value
Model 1 (R^2^=80.7%)			
MVPA (min/day)	80.3	0.777	<0.001
Sedentary time (min/day)	-5.1	-0.131	0.002
Body fat (%)	-35.3	-0.127	0.001
Model 2 (R^2^=80.5%)			
MVPA (min/day)	80.2	0.775	<0.001
Sedentary time (min/day)	-5.1	-0.132	0.001
Waist circumference (cm)	-26.6	-0.117	0.001
Model 3 (R^2^=81.0%)			
MVPA (min/day)	80.3	0.776	<0.001
Sedentary time (min/day)	-5.3	-0.136	0.001
Body mass index (kg/m^2^)	-76.3	-0.135	<0.001
**Girls**	**Non-standardized coefficient β**	**Standardized coefficient β**	**p-value**
Model 1 (R^2^=83.3%)			
Parents’ educational level (reference: incomplete high school)			
Complete high school	589.6	0.129	0.004
Undergraduation or graduation	210.9	0.039	0.399
MVPA (min/day)	101.8	0.837	<0.001
Sedentary time (min/day)	-3.8	-0.112	0.006

Multiple regression models adjusted for age and skin color - dependent variable: number of steps/day; excluded variables (p>0.05); boys: variables excluded in each model (p>0.05): quality of sleep, number of physical education classes; girls: family income, father’s professional status, number of siblings, number of televisions at home, television in the bedroom, school with physical activity policies, type of transport to school; MVPA: moderate to vigorous physical activity; min: minutes.

Due to the multicollinearity problems between BF, WC, and BMI (correlations greater than 0.90, and VIF>10) in boys, these variables were not included simultaneously in a single regression model. Given they are strongly associated to the number of steps, three regression models were conducted, each with one of these variables added to the remaining variables that had p<0.10 in the simple models (Table 5).

In boys, MVPA was positively related to the number of steps. On the other hand, ST, BF, WC, and BMI were negatively associated. In each model, the independent variables explain more than 80% of the number of steps/day. In girls, the combined educational level of parents and MVPA were related associated to the number of steps, and ST was negatively related. These variables, together, explained 83.3% of the number of daily steps (Table 5).

## DISCUSSION

The aim of the present study was to identify the behavioral and environmental indicators associated to the number of steps/day in children. With a significant difference (p<0.001), the mean steps/day for boys and girls were 10,470.57 and 8,573.23, respectively. Of all the variables analyzed in the multiple models, MVPA, ST, BF, WC, and BMI were significantly associated to the number of steps/day of boys. For girls, the educational level of their parents, MVPA, and ST were associated to the number of steps/day.

The average number of steps/day of the children participating in the study was lower than the WHO recommendations and the average of children from high-income countries.^5^ ^,^ ^6^ Knowing the importance of PA as a way of protecting children’s health, such data are highly worrying given the epidemic of physical inactivity and childhood obesity.^22^ ^,^ ^23^

Negative associations were found between the number of steps/day and BF, WC, and BMI in boys, but the same did not happen for goys. The number of steps/day is an important marker against childhood obesity and can also be used as an intervention to reduce the metabolic risk in children, even though it has a greater impact on boys than on girls.^24^ ^,^ ^25^ Other studies that related body composition with PA also found no association with girls.^25^ ^,^ ^26^

In both sexes, the number of steps/day was positively associated to MVPA, and negatively, to ST. Although treated as independent variables, there is a negative relationship between PA and ST.^27^ These results suggest that both ST and steps/day can be considered when strategic planning and program implementation are prepared to reduce risks in children.

Girls whose parents had completed high school had a higher number of steps/day than those whose parents did not complete high school. The educational level and PA of parents can have a great influence on the number of steps/day of their children. Craig et al.^28^ showed that the increase in the number of steps taken by parents was associated to an increase in the number of steps taken by their children: an increase of 1,000 steps/day for the father or mother determined the increase of 195-479 steps/day for their children. These findings highlight the influence of parents on the number of their children’s steps, especially in low and middle income countries, where the influence of parents’ educational level seems to have a greater impact on PA and children’s overweight.^29^ Higher educational level may be related to better socioeconomic conditions, thus allowing the choice for private spaces to practice PA when there is a lack of adequate public space.

The use of active transport can directly contribute to the number of steps/day.^30^ However, no significant associations were observed in the present study, which may be due to the short distance traveled from home to school by children who used active transport. Pabayo et al.^30^ did not observe either any significant associations between active transport and number of steps/day, but pointed out that those who used active transport to school were more likely to achieve step recommendations when compared to those who did not. Participation in physical education classes could also be related to the number of steps/day, with no associations found. We hypothesized that children are spending more time sitting than in movement in physical education classes, and, perhaps, this would justify such findings.

Some limitations must be considered. The study’s transversal design does not allow establishing a cause and effect relationship; sample is not representative; São Caetano do Sul City has a high HDI, besides PA and healthy eating policy and practice programs that help decrease ST and obesity of children in the city.^12^ ^,^ ^17^ ^,^ ^27^ In contrast, the use of an accelerometer as an instrument for objective measurement of the number of steps, in addition to considering the vast amount of lifestyle, and home and school environment variables, were certainly study’s strengths.

Further studies are needed to better understand the lifestyle indicators and the children’s environment that influence their number of steps/day. Because it is easily measured by several cell phone applications, other studies on this topic should be carried out. In addition, as it is an accessible and easy-to-use measure for children, encouraging them to increase the number of steps can be an important public health strategy. Although recent literature prioritizes studies on PA based on intensity and sedentary behavior, the authors of the present study highlight the importance of studying PA with steps/day, given that it is a basic movement of human locomotion, with numerous advantages aforementioned, besides having significant relations with health.^2^ ^,^ ^4^

Lifestyle indicators, body composition variables, and parents’ educational level were associated to their children’s number of steps/day. In boys, MVPA, ST, BF, WC, and BMI were associated to their number of steps/day. In girls, their parents’ educational level, MVPA, and ST were associated to their number of steps/day. Understanding the determinants of the number of steps/day can guide future interventions in children’s PA. Brazil remains with the challenge of promoting PA in the school community and other health indicators in Brazilian children.
